# Development of a 3-MicroRNA Signature and Nomogram for Predicting the Survival of Patients with Uveal Melanoma Based on TCGA and GEO Databases

**DOI:** 10.1155/2022/9724160

**Published:** 2022-11-22

**Authors:** Jun Zuo, Hongquan Ye, Jing Tang, Jianqun Lu, Qi Wan

**Affiliations:** ^1^Department of Ophthalmology, First People's Hospital of Linping District, Hangzhou, China; ^2^Department of Ophthalmology, The People's Hospital of Leshan, Leshan, China

## Abstract

**Background:**

The aim of this study was to apply bioinformatic analysis to develop a robust miRNA signature and construct a nomogram model in uveal melanoma (UM) to improve prognosis prediction.

**Methods:**

miRNA and mRNA sequencing data for 80 UM patients were obtained from The Cancer Genome Atlas (TCGA) database. The patients were further randomly assigned to a training set (*n* = 40, used to identify key miRNAs) and a testing set (*n* = 40, used to internally verify the signature). Then, miRNAs data of GSE84976 and GSE68828 were downloaded from Gene Expression Omnibus (GEO) database for outside verification. Combining univariate analysis and LASSO methods for identifying a robust miRNA biomarker in training set and the signature was validated in testing set and outside dataset. A prognostic nomogram was constructed and combined with decision curve as well as reduction curve analyses to assess the application of clinical usefulness. Finally, we constructed a miRNA-mRNA regulator network in UM and conducted pathway enrichment analysis according to the mRNAs in the network.

**Results:**

In total, a 3-miRNA was identified and validated that can robustly predict UM patients' survival. According to univariate and multivariate cox analyses, age at diagnosis, tumor node metastasis (TNM) classification, stage, and the 3-miRNA signature significantly correlated with the survival outcomes. These characteristics were used to establish nomogram. The nomogram worked well for predicting 1 and 3 years of overall survival time. The decision curve of nomogram revealed a good clinical usefulness of our nomogram. What's more, a miRNA-mRNA network was constructed. Pathway enrichment showed that this network was largely involved in mRNA processing, the mRNA surveillance pathway, the spliceosome, and so on.

**Conclusions:**

We developed a 3-miRNA biomarker and constructed a prognostic nomogram, which may afford a quantitative tool for predicting the survival of UM. Our finding also provided some new potential targets for the treatment of UM.

## 1. Introduction

Uveal melanoma (UM) is a highly aggressive form of ocular tumor in adult that usually derives from uveal melanocytes. Despite the incidence of UM is very low (about 0.06–0.07%), up to 50% of UM patients occur systemic metastases [1, 2]. The liver, the lung, and soft tissues are the most frequent metastatic sites. Currently, radiotherapy, chemotherapy, and enucleation are widely used for the treatment of UM and to prevent tumor recurrence. However, there are no effective therapies for metastatic UM, and the five-year survival rate of metastatic UM is very low [3, 4]. Unlike cutaneous melanoma, the most common mutations in UM are GNAQ, BAP1, and GNA11 [5]. Furthermore, UM metastases are less responsive to novel treatments such as immune checkpoint inhibitors and chemotherapies [6]. Currently, the best treatment remains uncertain, and the mechanisms underlying the prognosis of UM are not well illustrated. Therefore, identification of novel prognostic factors for therapy targets and clarify the survival events of UM are important.

Studies of mammalian transcriptional sequences indicated that only 1.5% of the human genome encoding protein. While about 70% of human genome is the noncoding RNAs [7]. MicroRNA is a class of noncoding RNA molecule, which at length of 19–25 nucleotides. It takes part in regulating the post-transcriptional expression of target mRNAs [8]. Recently, there are increasing evidences manifested that microRNA plays an important role in cell growth, development, invasion, differentiation, and apoptosis. Massive studies have proven that the aberrant expression of microRNA and its underlying molecular mechanisms were widely involved in tumorigenesis [9]. Moreover, previous studies showed that a complex microRNA regulatory network, rather than an individual microRNA, is involved in the regulation of metastasis of many cancers [10]. Some studies have revealed that microRNAs are associated with UM. For example, Zhou et al. prior reported that microRNA-20a acts as an oncogenic microRNA to promote tumor cells growth and movement in UM [11]. A recent study of microRNA also suggested that microRNA-34a can suppress UM cell proliferation and migration [12]. Thus, it is reasonable to believe that microRNAs may be considered as prognostic biomarkers.

The LASSO algorithm is a system biology-based approach. Compared with the traditional analysis for differential expression of genes, LASSO performs better at integrating information at both the expression of genes and the network topology level, which is widely used in cancer biomarker research and the identification of meaningful genes [13]. Additionally, a nomogram represents a mathematical model that combined plenty of important factors to predict a particular endpoint. For instance, the nomogram can combine clinical and pathological factors to estimate the probability of patients' risk of relapse and death [14, 15]. Hence, these approaches can be used to predict clinical prognosis and guide diagnosis and treatment.

Therefore, in this study, we used univariate analysis and the LASSO method to identify a robust microRNA biomarker [16]. According to the results of univariate and multivariate analyses for microRNA biomarker and clinical factors, a nomogram was established. To investigate the possible regulation of microRNA biomarkers, a microRNA-mRNA network was constructed. GO and KEGG pathway enrichment of all mRNAs in the network were performed. Our present study not only identifies a potential microRNA biomarker but also constructs a nomogram to better predict the survival of UM patients.

## 2. Materials and Methods

### 2.1. RNA Data and Clinical Characteristics

The RNA-sequencing data of microRNA and mRNA as well as clinical characteristics were extracted from The Cancer Genome Atlas (TCGA) of UM. MicroRNA expression profiles of GSE84976 and GSE68828 were obtained from the GEO database. Next, the 80 UM samples in the TCGA were equally classified into the training and testing datasets at random. The training dataset was used to identify key potential microRNA signatures. Then the testing dataset and the GSE84976 dataset were used for internal and external validate, respectively. Besides, the GSE68828 dataset contained 10 UM samples, including six monosomy 3 samples and four disomy 3 samples, which were used to explore the differential expression of signatures.

### 2.2. Development and Validation of MicroRNA Signature

To explore the associations between microRNA and the overall survival of UM patients, univariate cox regression was applied to select the potential prognostic microRNAs and mRNAs (*p* value <0.05). Afterwards, the LASSO method was used to develop prognostic model with these prognostic microRNAs. Based on the contribution of each variable, LASSO method weights each expression level of microRNA and selects the more favorable microRNAs to construct risk system model and calculates coefficients. The risk model computes a detailed risk score for all patients, which was further divided into high- and low-risk groups. Their clinical characteristics about stage and age were also divided into subgroups. The different survival curve among groups were illustrated by the Kaplan–Meier methods and combined with the log-rank test. Besides, to test the model performance, the receiver operating characteristic (ROC) curve and area under the receiver operating characteristic curve (AUC) value were generated to estimate the specificity and sensitivity of the model [17].

### 2.3. Construct the Nomogram

The factors analyzed in this study are as follows: age, gender, TNM classification, stage, and microRNA signature. The relationships between microRNA signature expression and clinical characteristics were also performed. The univariate and multivariate cox regressions were conducted to estimate the influence of factors on overall survival (OS). The hazard ratio (HR) was used to estimate the influence of each factor on OS. The significant variables acquired from the univariate and multivariate cox regressions were used to construct the nomogram. A ROC curve analysis was applied to predict the accuracy of the nomogram model. Besides, a decision curve and a reduction curve were performed to estimate the clinical usefulness of the nomogram model.

### 2.4. Prediction of MicroRNA-mRNA Interactions and Construction of Network

First, the microRNA-mRNA interactions data were obtained from some trustworthy microRNA reference database including miRTarBase (https://mirtarbase.mbc.nctu.edu.tw/php/index.php), miRDB (https://www.mirdb.org/), and TargetScan (https://www.targetscan.org/vert_71/) [18]. Second, the microRNA-mRNA pairs were predicted using these three databases and combining with survival related mRNAs. Finally, we established matched microRNA-mRNA network. The microRNA-mRNA network was visualized by using Cytoscape 3.6.0.

### 2.5. Pathway Enrichment Analysis

The functions of selected microRNA-paired mRNA were assessed by the biology process (BP) term in gene ontology (GO) enrichment analysis and the Kyoto Encyclopedia of Genes and Genomes (KEGG) enrichment analysis. With the analysis of these microRNA-paired mRNAs in the context of biological domain knowledge, the biological functions associated with the molecular network can be comprehensively understood. Pathways with a *p* value <0.05 were regarded as significant.

### 2.6. Statistical Analyses

All statistical tests were conducted using the R software (version 3.5.2). The Cox regression was estimated in TCGA by the “survival” package. The LASSO statistical algorithm was conducted on TCGA and GEO datasets by the “glmnet” package. The nomogram was constructed in TCGA by the “rms” package. Kaplan–Meier curves and ROC curves were drawn in the TCGA and GEO datasets by the “survival” and “survivalROC” packages, respectively. Differences in clinicopathological characteristics between the training and testing cohorts were analyzed using the *t*-test or chi-square test. *P* < 0.05 was considered as the significant threshold in all statistical tests and 95% confidence interval (CI) also estimated.

## 3. Results

### 3.1. Processing of MicroRNAs and mRNAs

After excluding tinier expression level, 15,187 mRNAs and 1581 microRNAs in 80 UM patients were acquired from TCGA after these steps. Next, these patients were randomly separated into a training dataset (*n* = 40, used to identify key miRNAs) and a testing dataset (*n* = 40, used to internally verify the signature). The clinical characteristics of the training and testing dataset are listed in Table 1. The statistical results suggested that no significant differences existed between the two datasets.

### 3.2. Development and Validation of MicroRNA Signature

**Figure 1:**
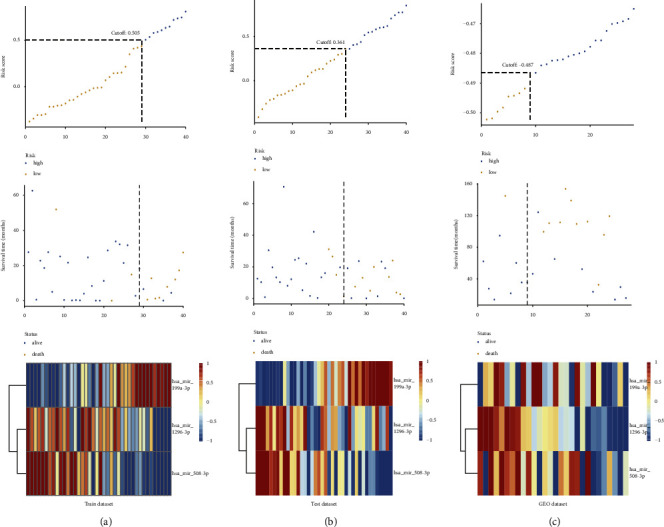
Risk score analysis of the training (*n* = 40), testing (*n* = 40) and GSE84976 datasets. (a): The distribution of risk score, patients' survival time and status, heatmap of 3-miRNA expression signature in training dataset. (b): The distribution of risk score, patients' survival time and status, heatmap of 3-miRNA expression signature in testing dataset. (c): The distribution of risk score, patients' survival time and status, heatmap of miRNA expression signature in GSE84976 dataset.

**Figure 2:**
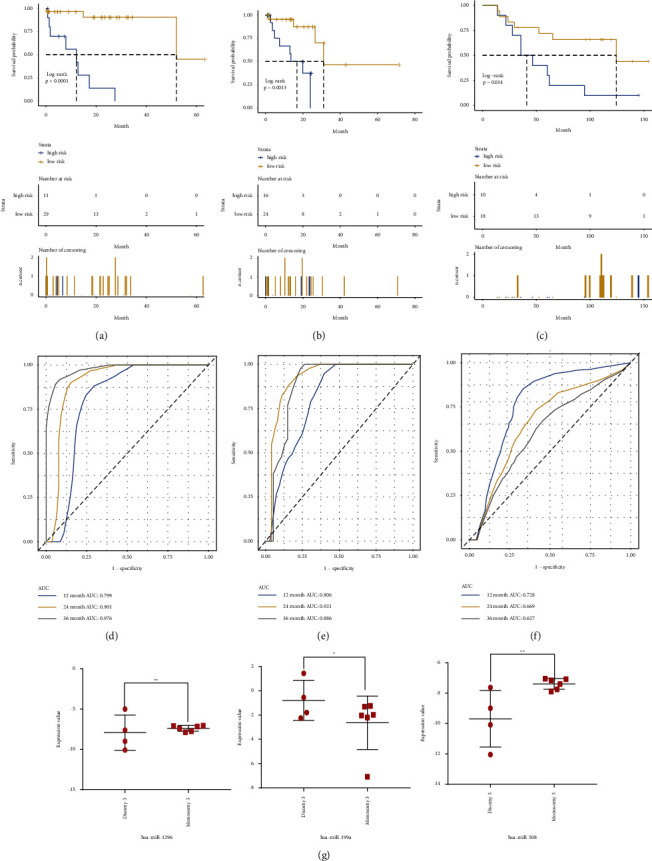
Kaplan–Meier curve survival analysis for 3-miRNA prognostic signature of uveal melanoma (UM). (a–c): The Kaplan–Meier curve of the overall survival (OS) between the high-risk and low-risk groups stratified by the median risk score in training, testing and GSE84976 datasets respectively. (d–f): The receiver operating characteristic (ROC) curves of 3-miRNA in training, testing and GSE84976 datasets, respectively. (g): The plot analysis of 3 miRNAs in GES68828 dataset. ^*∗*^*P* < 0.05, ^*∗∗*^*P* < 0.01.

We first excluded the nontumorous causes of death in this study. Next, we performed univariate regression analysis and LASSO modelling to assess relationships between microRNAs and the overall survival (OS) time of UM in the training dataset. Finally, a 3-microRNA biomarker was identified from 581 microRNAs. The LASSO generated coefficients for 3 microRNAs and the risk score formula as follows: −0.0596 × (expression value of has-miR-1296-3p) + 0.1062 × (expression value of hsa-miR-199a-3p) + −0.0461 × (expression value of hsa-miR-508-3p). The risk score of patients was calculated using a risk score formula. Moreover, patients were classified into high- and low-risk groups by using the optimal cut-off of risk scores. The vital status, risk score distributions, and expression value of three microRNAs in the training, testing, and GSE84976 datasets were presented in Figure 1. Based on the heatmap of expression value of three microRNAs, we observed that hsa-mir-199a-3p was upregulated in advanced tumors, while has-mir-1296-3p and hsa-mir-508-3p were downregulated in advanced tumors. Kaplan–Meier curves manifested that UM patients in high risk have a shorter survival time than those in low risk with a log-rank test *p* < 0.0001 (Figure 2(a)). To demonstrate the predictive ability of the 3-microRNA biomarker, the same 3 microRNAs in testing and GSE84976 datasets were used to verify the results. The patients in the testing and GSE84976 datasets were classified into high-andlow-risk groups based on the training dataset (Figures 1(b) and 1(c)). Kaplan–Meier curves manifested that high risk patients have a worse prognosis than those in low risk both in testing and GSE84976 datasets (log-rank *p*=0.0013 and *p*=0.014) (Figures 2(b) and 2(c)). The ROC curves were applied to estimate the prediction power of microRNA in training, testing, and GSE84976 datasets (Figures 2(d)–2(f)). Furthermore, the plot analysis of 3 microRNAs in the GSE68828 dataset indicated that the expression of 3 microRNAs had significant differences between monosomy 3 samples and disomy 3 samples (Figure 2(g)).

### 3.3. Construct the Nomogram

**Figure 3:**
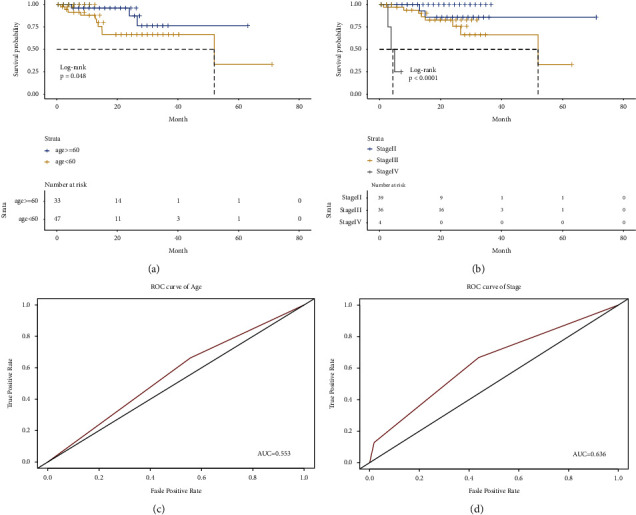
Kaplan–Meier survival analysis of age and stage (a, b): Kaplan–Meier survival analysis for age and stage prognostic signature of uveal melanoma (UM). (c, d): The ROC analysis was used for the discrimination between subgroup.

**Figure 4:**
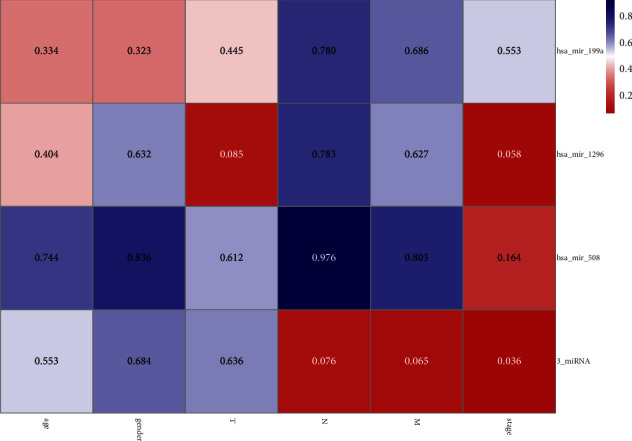
The heatmap of miRNAs signature expression and clinical characteristics (the blue and the red colors represent higher and lower *P* value, respectively).

**Figure 5:**
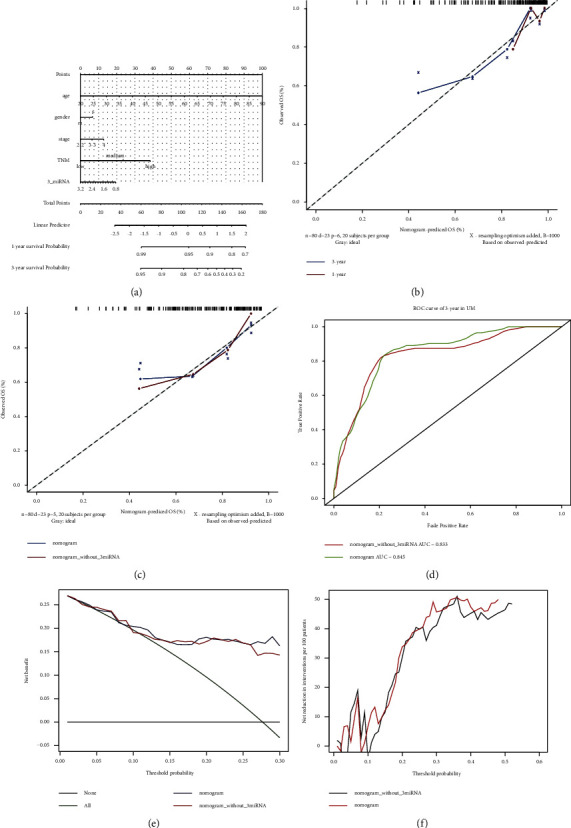
Nomogram construction for predicting overall survival in uveal melanoma. (a): The nomogram composed by the 3-miRNA score, stage, TNM, age, and gender. The total points were calculated by drawing a vertical line from variable values to the axis labelled “Points” which can predicted 1 and 3 years of overall survival (OS). (b): Calibration curves of the nomogram for predicting OS at 1 year (red solid line) and 3 years (blue solid line). (c): Calibration curves of the nomograms for predicting OS in without 3-miRNA model (red solid line) and 3-miRNA model (blue solid line). (d): ROC curve for the accuracy of nomogram. The whole nomogram (green solid line) had higher accuracy than the nomogram model without 3-miRNA risk score (red solid line). (e): Decision curve of the nomogram for 3 years of OS. The green line means the speculation that all patients survive in the 3 years. The gray line means the speculation that no patients survive in the 3-year. The red and blue line means the nomogram without 3-miRNA model and integrated nomogram model respectively. (f): The net reduction curves of the nomogram without 3-miRNA model (black solid line) and nomogram model (red solid line).

The univariate and multivariate cox regressions were conducted to estimate prognostic factors for OS (shown in Table 2). Age (*P*=0.020), stage (*P*=0.003) were significantly associated with OS. Kaplan–Meier curves showed that patients with old age (≥ 60 years) and late tumor stage had a significantly poor OS (Figures 3(a) and 3(b)). The AUCs of age and stage were 0.553, 0.636, respectively (Figures 3(c) and 3(d)). The heatmap of microRNAs signature expression and clinical characteristics indicated that 3-microRNA biomarker was significantly associated with stage (Figure 4). Factors considered significant in the univariate and multivariate cox analyses were enter in the nomogram construction. Finally, age, stage, TNM classification, and 3-microRNA biomarker were incorporated in the nomogram. Afterwards, the points of each parameter were summarized a total point which can assess the 1 and 3 years of survival probabilities (Figure 5(a)). The calibration curves of nomogram revealed that a good consistency exists between prediction and actual survival (Figures 5(b) and 5(c)). The AUC of nomogram model have a higher accuracy than the without 3-microRNA model (Figure 5(d)). Eventually, in order to estimate whether the nomogram was clinically useful, decision curve and reduction curve analyses were conducted to evaluate the net benefit and reduction of the models. Compared with the without 3-microRNA nomogram model, the overall nomogram model provided the better clinical utility (Figures 5(e) and 5(f)).

### 3.4. Prediction of MicroRNA-mRNA Interactions and Construction of Network

**Figure 6:**
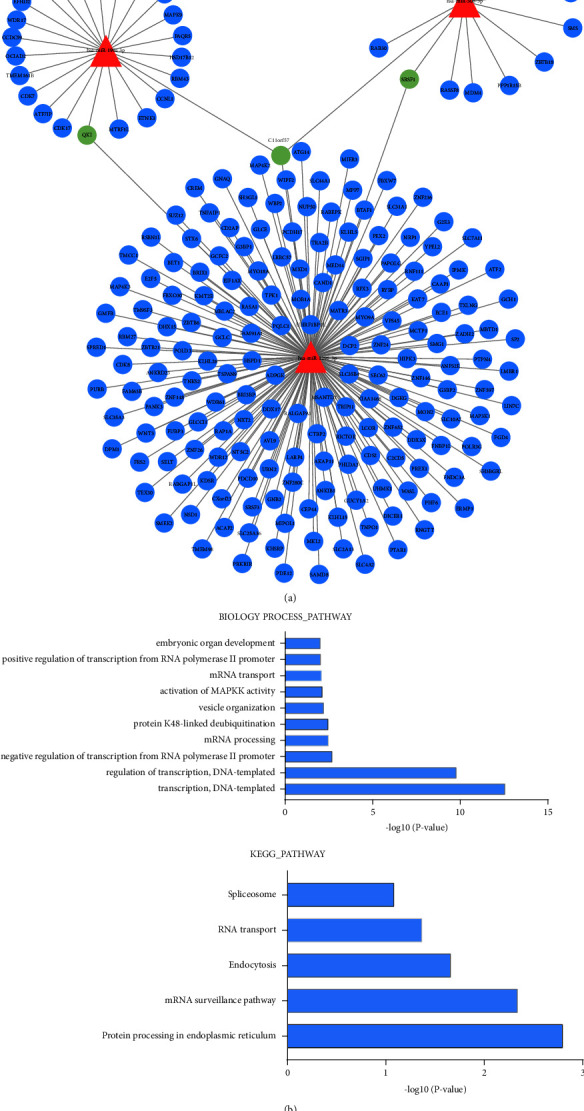
The miRNA-mRNA network. (a): The miRNA-mRNA network is composed of 3 miRNAs and 218 mRNAs. Triangles and circles represent miRNAs and mRNAs, respectively. The green circles represent genes shared by two or more miRNAs. (b): The top ten BP term in GO and KEGG functional pathway enrichment analysis.

Totally, 221 pairs of microRNA-mRNA network were constructed in UM it was composed of 3 microRNAs and 218 mRNAs. The network presented in Figure 6(a).

### 3.5. Pathway Enrichment Analysis

In all, 217 microRNA-paired mRNAs were used to performed BP term and KEGG pathway enrichment analyses. The results of BP revealed that these paired mRNAs were significantly enriched in biological functions associated with regulation of transcription DNA-template, mRNA processing, mRNA transport, and so on (The top ten shown in Figure 6(b)). The KEGG enrichment showed that paired mRNAs were significantly enriched in pathways such as the spliceosome, RNA transport, endocytosis, and so on (Figure 6(b)).

## 4. Discussion

The increasing genome-wide researches proven that majority of the cellular genomes are transcribed, and there is a complex RNA network which contains lots kind of RNA molecules. But only around 2% of the transcripts own the ability to translate proteins. Recent studies have demonstrated that microRNAs take crucial regulatory roles in many biological processes of human tumor including UM [19–23]. With the advances of sequencing techniques, growing computational studies aimed at identifying microRNAs biomarkers for the diagnosis and prognosis of cancers due to their regulation of target gene expression [24–28]. Furthermore, recent evidences suggested that the abnormal expression of microRNAs significantly correlated with the prognosis of UM patients and could be regarded as a potential target for treatment, like the previous demonstrated microRNAs: hsa-miR-374b-5p, hsa-miR-29c-3p, and hsa-miR-211-5p [29]. However, some researchers questioned that it is not sufficient for these molecules to accurately predict the prognosis of patients. Because they failure to take the simultaneous change of multiple microRNAs and clinical information into account. Therefore, to explore the prognostic value of microRNAs in UM, we conducted univariate analysis and the LASSO algorithm to identify the microRNAs biomarker, which has a significantly positive correlation with the OS of UM in the training set, testing dataset, and outside dataset. Then we further used the microRNAs biomarker and associated it with clinical characteristics to build a nomogram model, which manifested a good survival prediction for UM.

In this research, we distinguished a 3-microRNA biomarker to predict the prognosis of UM in the TCGA dataset and found these microRNAs have significant differences between monosomy 3 and disomy 3 samples in the GEO dataset. Many research studies have also suggested that UM with monosomy 3 is closely correlated with a dramatically poor prognosis. Therefore, it seems rational to speculate that the alternation of 3-microRNA biomarkers will cause the mutation of chromosomal 3 and finally lead to a poor prognosis. In order to increase the prediction accuracy of UM, the 3-microRNA biomarker combined with clinical characteristics was incorporated in univariate and multivariate cox regression. The results indicated that age (*P*=0.020), stage (*P*=0.003), 3-microRNA biomarker (*P*=0.032) were significantly in associated with OS. The result of Kaplan–Meier analyses indicated that patients with old age (≥ 60 years) and at the high risk of 3-microRNA in late-stage will have a poor prognosis. Stratified analysis revealed that this 3-microRNA biomarker was proper for predicting the stage of UM (Figure 4, *P*=0.036). The nomogram, which can associate multiple biological variables with clinical factors to calculate the probability of clinical events [30–32], we constructed a new nomogram depended on 80 UM patients in the TCGA database, which can predict the three years overall survival rates of UM. Calibration curves demonstrated that the nomogram for predicting 1 year and 3 years of survival time was close to the ideal prediction model. Moreover, compared with the 3 microRNA model, the calibration curve including 3-microRNA biomarker was closer to the ideal line and can better predicting 3-year OS [33]. Moreover, compared with the without 3-microRNA nomogram model, the overall nomogram model had higher accuracy and offered the better clinical utility. Therefore, our nomogram can afford a simpler and more accurate tool to predict the prognosis of UM. The established microRNA signature that can distinguish categories of UM patients who may benefit from chemotherapy or combination therapies. The use of the nomogram to assess UM patients in clinic offers up new possibilities for the future.

To better understand the molecular functions of the three microRNAs, we constructed a microRNA-mRNA network to predict their target mRNAs. KEGG pathways and GO enrichment analysis of target mRNAs revealed that these paired genes were significantly enriched in the spliceosome pathway, the RNA/mRNA transport pathway, the regulation of transcription DNA-template, and so on. The results suggested that these microRNAs might take part in transcriptional and splicing regulation pathways affect the occurrence and development of UM. It has been proven that spliceosoma mutations not only exist in patients with leukemia or myelodysplastic syndrome, but can also occur in some solid tumors, including breast cancers, lung cancers, and uveal melanoma [34–36]. Their presence in a variety of malignant tumors indicates that splicing and transcriptional mutations may play an important role in the definition of malignant phenotypes [37–39]. Therefore, we speculated that these microRNAs were regarded as the most important role in the prognosis of UM.

Despite the fact that we found some significant prognostic microRNAs and built a nomogram to predict the survival of UM, our study has several limitations. Firstly, our study is according to bioinformatic analysis, and experiments in in vitro and in vivo are lacking. Additionally, the sample size of this study is small, and the race only included Caucasians. As a result, more research into the underlying molecular process will be required.

## 5. Conclusions

In summary, our study highlighted a 3-microRNA biomarker and nomogram for predicting the survival of patients, which might be regarded as new promising biomarkers for UM prognosis and treatment.

## Figures and Tables

**Table 1:** Clinicopathological characteristics of training and testing datasets. *T*: tumor; *N*: node; *M*: metastasis; SD: standard deviation.

	Testing datasets	Training datasets	*p* values
*n*	40	40	
Vital_status = DEAD (%)	12 (30.0)	11 (27.5)	1
Race = white (%)	29 (100.0)	26 (100.0)	
Age (mean (SD))	66.98 (13.86)	60.59 (14.28)	0.046
Gender = MALE (%)	25 (62.5)	20 (50.0)	0.367
Stage (%)			0.627
	0 (0.0)	1 (2.5)	
IIA	6 (15.0)	6 (15.0)	
IIB	11 (27.5)	16 (40.0)	
IIIA	13 (32.5)	12 (30.0)	
IIIB	6 (15.0)	4 (10.0)	
IIIC	1 (2.5)	0 (0.0)	
IV	3 (7.5)	1 (2.5)	
*M* (%)			0.492
m0	26 (66.7)	25 (64.1)	
m1	2 (5.1)	0 (0.0)	
m1b	1 (2.6)	1 (2.6)	
mx	10 (25.6)	13 (33.3)	
*N* = nx (%)	13 (33.3)	14 (35.0)	1
*T* (%)			0.498
t2a	6 (15.0)	6 (15.0)	
t2b	0 (0.0)	2 (5.0)	
t3	0 (0.0)	1 (2.5)	
t3a	11 (27.5)	14 (35.0)	
t3b	2 (5.0)	3 (7.5)	
t3c	0 (0.0)	1 (2.5)	
t4a	12 (30.0)	8 (20.0)	
t4b	6 (15.0)	3 (7.5)	
t4c	1 (2.5)	1 (2.5)	
t4d	2 (5.0)	0 (0.0)	
t4e	0 (0.0)	1 (2.5)	
Age_group = younger (%)	17 (42.5)	23 (57.5)	0.264
Time (mean (SD))	15.45 (13.89)	14.96 (15.23)	0.88

**Table 2:** Univariate and multivariate cox regression of clinical characteristics associated with overall survival in TCGA dataset.

	Univariate analyses				Multivariate analyses			
	unicox_*p* values	HR	Low	High	mutlicox_*p* value	Exp (coef)	Lower: 95	Upper: 95
Age	0.0205^*∗*^	1.058	1.009	1.109	0.007^*∗*^	1.089	1.024	1.158
Gender	0.354	1.747	0.537	5.686	0.531	1.517	0.411	5.596
Stage	0.004^*∗*^	8.450	1.990	35.886	0.527	1.994	0.235	16.909
*m*	0.024^*∗*^	7.359	1.302	41.579	0.182	4.151	0.514	33.536
*n*	0.086	0.364	0.115	1.155	0.010^*∗*^	0.039	0.003	0.455
*t*	0.239	1.831	0.668	5.013	0.485	1.880	0.319	11.083
3-miRNA	0.414	0.574	0.151	2.178	0.032^*∗*^	0.182	0.038	0.867

## Data Availability

The datasets generated for this study can be found in the GEO databases (GSE84976 and GSE68828; https://www.ncbi.nlm.nih.gov/geo/) and UCSC Xena website (TCGA-UVM; https://gdc.xenahubs.net).

## Abbreviations:

TCGA: The cancer Genome Atlas
GEO: Gene Expression Omnibus
UM: Uveal melanoma
GO: Gene ontology
KEGG: Kyoto Encyclopedia of Genes and Genomes
OS: Overall survival
AUC: The area under the curve
ROC: Receiver operating characteristic curve.

## Additional Points

A preprint has previously been published: DOI:10.21203/rs.3.rs-572728/v1 [33].
